# Retention in care and viral suppression in differentiated service delivery models for HIV treatment delivery in sub‐Saharan Africa: a rapid systematic review

**DOI:** 10.1002/jia2.25640

**Published:** 2020-11-28

**Authors:** Lawrence Long, Salome Kuchukhidze, Sophie Pascoe, Brooke E Nichols, Matthew P Fox, Refiloe Cele, Caroline Govathson, Amy N Huber, David Flynn, Sydney Rosen

**Affiliations:** ^1^ Department of Global Health Boston University School of Public Health Boston MA USA; ^2^ Health Economics and Epidemiology Research Office Department of Internal Medicine School of Clinical Medicine Faculty of Health Sciences University of the Witwatersrand Johannesburg South Africa; ^3^ Alumni Medical Library Boston University Boston MA USA

**Keywords:** antiretroviral therapy, differentiated service delivery, retention, suppression, Africa, systematic review

## Abstract

**Introduction:**

Differentiated service delivery (DSD) models for antiretroviral treatment (ART) for HIV are being scaled up in the expectation that they will better meet the needs of patients, improve the quality and efficiency of treatment delivery and reduce costs while maintaining at least equivalent clinical outcomes. We reviewed the recent literature on DSD models to describe what is known about clinical outcomes.

**Methods:**

We conducted a rapid systematic review of peer‐reviewed publications in PubMed, Embase and the Web of Science and major international conference abstracts that reported outcomes of DSD models for the provision of ART in sub‐Saharan Africa from January 1, 2016 to September 12, 2019. Sources reporting standard clinical HIV treatment metrics, primarily retention in care and viral load suppression, were reviewed and categorized by DSD model and source quality assessed.

**Results and discussion:**

Twenty‐nine papers and abstracts describing 37 DSD models and reporting 52 discrete outcomes met search inclusion criteria. Of the 37 models, 7 (19%) were facility‐based individual models, 12 (32%) out‐of‐facility‐based individual models, 5 (14%) client‐led groups and 13 (35%) healthcare worker‐led groups. Retention was reported for 29 (78%) of the models and viral suppression for 22 (59%). Where a comparison with conventional care was provided, retention in most DSD models was within 5% of that for conventional care; where no comparison was provided, retention generally exceeded 80% (range 47% to 100%). For viral suppression, all those with a comparison to conventional care reported a small increase in suppression in the DSD model; reported suppression exceeded 90% (range 77% to 98%) in 11/21 models. Analysis was limited by the extensive heterogeneity of study designs, outcomes, models and populations. Most sources did not provide comparisons with conventional care, and metrics for assessing outcomes varied widely and were in many cases poorly defined.

**Conclusions:**

Existing evidence on the clinical outcomes of DSD models for HIV treatment in sub‐Saharan Africa is limited in both quantity and quality but suggests that retention in care and viral suppression are roughly equivalent to those in conventional models of care.

## 1 Introduction

Throughout sub‐Saharan Africa, most national HIV programmes are striving to achieve the 95‐95‐95 targets for HIV diagnosis, treatment and viral suppression [1]. The rapid expansion of antiretroviral therapy (ART) programmes to reach these targets has created shortfalls in health system capacity and quality [2]. In response, many countries are scaling up alternative service delivery approaches, or differentiated service delivery (DSD) models. DSD models differ from conventional HIV care in the location and frequency of interactions with the healthcare system, cadre of provider involved, and/or types of services provided [3]. Grimsrud and colleagues [4] broadly categorize DSD models as individual or group models, with service delivery at a facility or in the community. DSD models aim to achieve a wide range of potential benefits to both providers and patients. The attractiveness of DSD models is generally considered to be conditional on maintaining at least equivalent clinical outcomes to conventional care; assuming no deterioration in clinical outcomes, DSD models are hoped to generate greater patient satisfaction, lower cost to both providers and patients and create efficient and convenient service delivery.

Despite the large‐scale rollout of DSD models in various formats across multiple countries, there is a dearth of evidence to document the purported benefits of the new models in routine implementation. Even the minimum requirement of equivalent clinical outcomes is poorly documented for most models and settings. The studies and evaluations available are widely inconsistent in their designs, methods and outcomes, making it difficult to draw an overall picture of the impact of the models. Monitoring and evaluation systems have not kept up with DSD model implementation, and DSD participation is poorly captured in routine records, making it challenging to compare outcomes in DSD models with those in conventional care [5]. The information available to policy makers, funders and programme implementers is thus incomplete and difficult to interpret.

To help fill this gap and create a baseline to guide future research, we conducted a rapid systematic review of the most recent peer‐reviewed reports of the outcomes of DSD model implementation in sub‐Saharan Africa. In view of the importance of achieving non‐inferior clinical outcomes as a condition for adopting DSD models, we report here the results of our search for retention in care, viral suppression and related clinical outcomes.

## 2 Methods

Following World Health Organization guidance for rapid reviews [6], we conducted a rapid systematic review of peer‐reviewed publications and conference abstracts that reported outcomes of differentiated service delivery (DSD) models for the provision of antiretroviral treatment (ART) in sub‐Saharan Africa since 2016 [7]. The search protocol was previously presented [7], and the review was registered on the International Prospective Register of Systematic Reviews (PROSPERO CRD42019118230).

Although the full review included a wide range of outcomes for both providers and patients, the most widely available information pertained to patient‐level clinical outcomes, specifically retention in care and viral suppression. In this report we focus on these outcomes only, to allow for a more detailed examination and discussion of consistently defined indicators. The full report of the review is available online [8].

### 2.1 Search strategy and study selection

For this review, we adopted and modified the widely‐cited frameworks put forward by Grimsrud *et al* [4] and Duncombe *et al* [3] and defined as a “differentiated model of service delivery” any approach to providing ART that focused on a specific population, the location of service delivery, the frequency of patient interaction with the healthcare system, or the cadre of healthcare provider involved [9]. We did not consider a change in services provided, without adjustment of any other characteristics, to constitute a DSD model. DSD models for all populations except for pregnant women in PMTCT programmes and clients on ART for HIV prevention (PEP or PrEP) were included in this review. A full list of inclusion and exclusion criteria for the review are shown in Table S1.

We searched the PubMed, Embase and Web of Science databases with a search string developed to identify publications which reported on HIV treatment delivery models in sub‐Saharan Africa from 1 January 2016 until 12 September 2019. The final search was conducted on 12 September 2019. We supplemented the peer‐reviewed publications by manually searching peer‐reviewed abstracts from major conferences for the same period. Search strings and a full list of conferences included can be found in Table S2. Limiting eligible articles and abstracts to those published or presented since 2016 was intended to ensure that results come as close as possible to reflecting the current state of DSD model implementation and to avoid repeating the efforts of previous reviews [2, 10, 11, 12, 13]. If a source reported patient follow‐up data collected both before and after January 1, 2016, we included it only if the majority of follow‐up time (more than 50%), as stated in the source or estimated by the authors, occurred after that date. Therefore, the bulk of the implementation period for the models included is 2016 or later.

We excluded sources that reported interventions aimed at improving conventional care that we judged did not in themselves comprise DSD models, such as adherence interventions that strengthened existing counselling or offered incentives for retention within the conventional model of care. We also excluded cross‐sectional surveys of patients or providers who were asked to comment on DSD models but did not have personal experience with it. If two source documents described what we determined to be the same cohort of patients enrolled in the same instance of the model, we counted only one model but cited both references for it. If one source document superseded another, for example by reporting more complete data or longer term outcomes, we kept only the more informative source. Where full conference presentations or posters were available, we used these rather than the abstracts. If two source documents reported data on the same study, we included the one with the most recent results.

All peer‐reviewed references identified using the respective search strings from PubMed, Embase and Web of Science were imported into an EndNote™ library, where deduplication occurred. An initial, independent, blinded review (reviewers were not aware of each other’s decisions) of the titles and abstracts was conducted by three study team members (SK, RC, CG) using Rayyan QCRI [14]. A full‐text review was then conducted for all publications remaining after the initial review by two study team members (SK, CG). Reasons for excluding publications were recorded during the full‐text review. As a quality check, another author (LL) also checked a sample (10%) of the excluded sources against exclusion criteria. At each stage of the review process, any conflicts between reviewers were assessed and resolved through the consensus of two authors (LL, SR). The results of the search were documented in accordance with the PRISMA‐P reporting checklist (Text S1) [15, 16].

### 2.2 Data extraction

The data extraction tool was designed to capture each DSD model separately, regardless of whether the source publication described one or many models. In addition to standard bibliographic descriptors, we collected two types of data: a) a detailed description of the model of service delivery; and b) the outcomes that were reported for the model. We categorized each model according to the taxonomy described by Grimsrud [4], with four categories: facility‐based individual models, out‐of‐facility‐based individual models, healthcare worker‐led groups and client‐led groups. We then used the adapted Duncombe [3] schema to describe the model in terms of population, provider, location, frequency and services provided as well as and its outcomes. Where a comparison was provided with the pre‐ or non‐differentiated standard of care, we also extracted data about these comparison models, henceforth referred to as conventional care.

### 2.3 Outcomes

We report here standard clinical HIV treatment metrics, including retention in care, viral load suppression, adherence and pharmacy refill rates. We used each source’s own definition and timing of these outcomes, accepting that definitions for “retention in care” vary widely, as do thresholds for determining viral suppression. Retention usually referred to the proportion of patients enrolled in a DSD model and retained in the ART programme at a specific time point after enrolment in the study. The point at which a patient was considered no longer in care (i.e. not retained) varied by study or country. Where a loss to follow‐up (LTFU) proportion was reported, we converted it to a retention rate (as 100‐LTFU%). Most sources defined viral suppression as <1000 copies/mL. Viral suppression was not always reported among those retained in care. Adherence and prescription refill frequency were uncommon outcomes but are included in this analysis when reported. Other outcomes from the full review, such as costs to providers and patients, can be found elsewhere. [17, 18]

### 2.4 Analysis

To structure the results, we first divided the models into the four categories mentioned above: facility‐based individual models (FBIM), out‐of‐facility‐based individual models (OFBIM), client‐led groups (CLG) and healthcare worker‐led groups (HCWLG). In publications where more than one model was described, we counted each model separately. We report outcomes as stated in the original publications, adjusted where possible to utilize uniform metrics (e.g. by converting a reported percentage of patients lost to follow‐up to the percentage of patients retained). As explained in the search protocol [7], we feared that it would be misleading to conduct aggregate analyses due to the heterogeneity of model designs, participating populations and study settings, even where outcomes themselves were similar. We thus report only the disaggregated results.

We assessed the quality of the cohort studies using the Newcastle‐Ottawa scale [19, 20]. The quality rating covered a review of selection, comparability and outcome domains and generated a score out of 9. There are no standardized quality rating categories, but to simplify interpretation of scores, those studies that scored 7 or above were categorized as high quality, those scoring between 4 and 6 were of moderate quality, and those scoring below 4 were considered low quality, as done in previous studies [20]. Randomized controlled trials were assessed using the Cochrane Collaboration’s tool for assessing the risk of bias in cluster randomized controlled trials [21]. We assessed sequence generation, participant recruitment with respect to randomization timing, deviation from intended intervention, completeness of outcome data for each main outcome, bias in the measurement of outcome, bias in the selection of the reported result. The risk of bias assessment for the one remaining cross‐sectional study was not conducted [22].

## 3 Results

### 3.1 Sources identified

**Figure 1:**
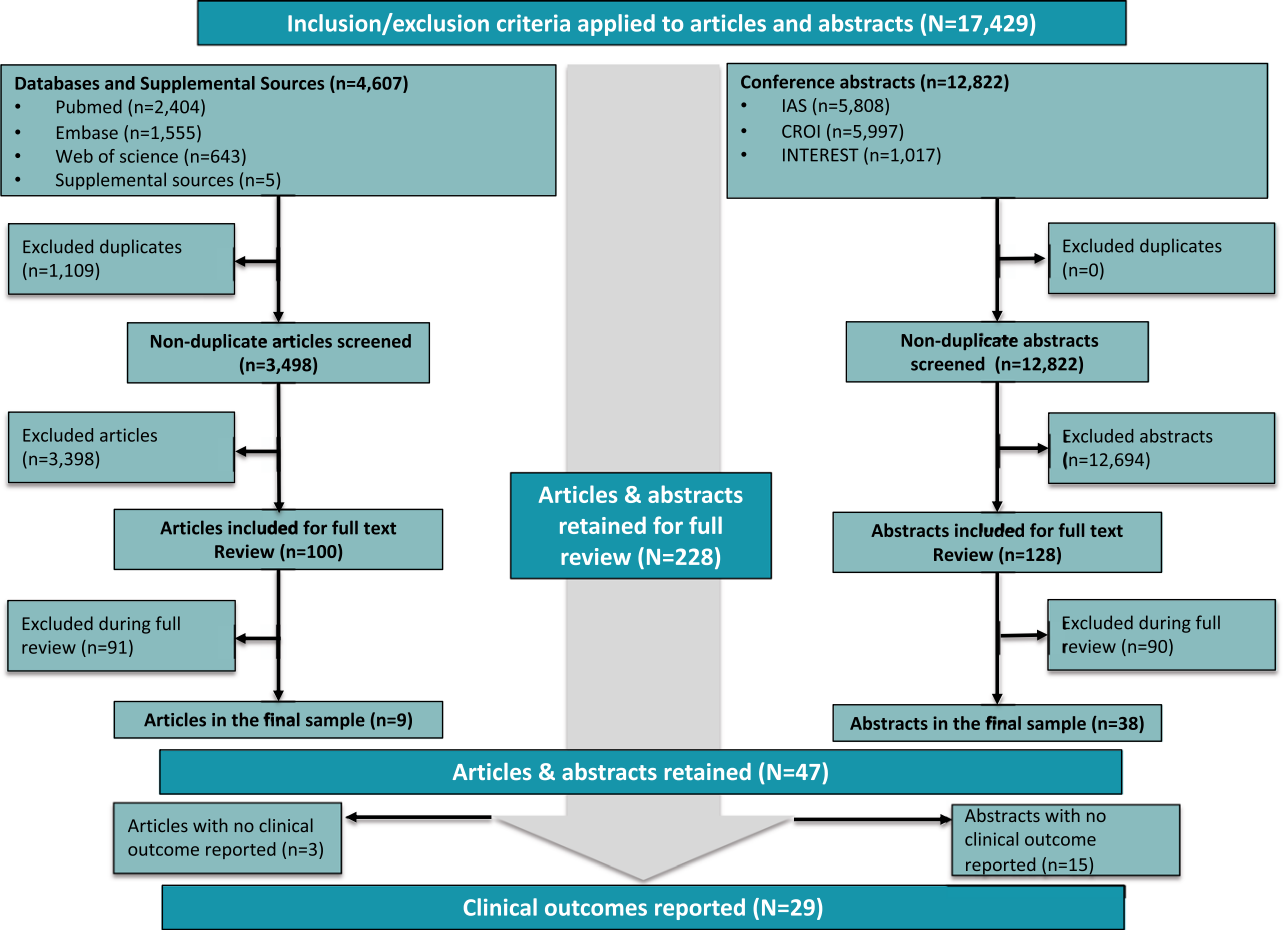
PRISMA flow chart. Conference abstracts accounted for the majority of references that were reviewed and ultimately included. 228 articles and abstracts were retained for full review, of which 29 were ultimately included and reported

The results of the systematic search are shown in Figure 1. A total of 3,498 non‐duplicate abstracts of peer‐reviewed journal articles and 12,822 abstracts from the selected conferences were screened. After the initial title and abstract review, 16,092 articles and abstracts were excluded, leaving 228 documents for full review. During the full review, an additional 181 were excluded. Reasons for exclusions are reported in Table S3. The primary reason (60%) for excluding articles was date: most or all of the underlying data were collected prior to 2016. The main reason for excluding conference abstracts (33%) was insufficient information to adequately describe the model and at least one of the outcomes of interest.

Nine peer‐reviewed articles and 38 conference abstracts (47 total) were retained in the final data set for the full review. Of these, 29 included one or more clinical outcomes and were included in the analysis reported here. Three quarters of these sources (76%) reported observational cohort studies; most of the rest (21%) were randomized trials. South Africa (27%) and Zambia (22%) jointly accounted for nearly half the sample (Table S3).

### 3.2 Differentiated models included in the review

The 29 sources described outcomes for a total of 37 discrete differentiated service delivery models, excluding conventional care models for comparison. Models are described briefly in Table 1 below and in full in Table S3. In the tables, each model is assigned a model identifier (ID), which is used to reference that model throughout the review. If a source document (article or abstract) reports on more than one DSD model, multiple model IDs will be associated with it in Table 2. Each model identifier contains an acronym for the model category (FBIM, OFBIM, CLG or HCWLG) followed by a number. For example client‐led groups have model IDs CLG1 through CLG5, indicating that there were five distinct CLG models identified. In one instance (HCWLG11), the same model is referred to in more than one source document [23, 24].

**Table 1:** Models included in the review

ID	Source	Country	Model	Eligibility for model (condition, age^a^)	Sample size^c^	Study period
**Facility‐based individual models (FBIM)**						
FBIM1 [25]	Kamerhe 2018	Democratic Republic of Congo (DRC)	Fast‐track ART refill circuit	Stable^d^, ≥18	974	10/2016 to 03/2018
FBIM2 [26]	Havlir 2019^b^	Kenya and Uganda	Patient‐centred streamlined care	No restriction, not specified	186,354	2013 to 2017
FBIM3 [27]	Mdala 2018	Namibia	Nurse‐initiated management of ART	No restriction, not specified	18,367	09/2015 to 09/2017
FBIM4 [28]	Cassidy 2018	South Africa	Quick pick‐up model	Stable, not specified	4,753	10/2015 to 10/2017
FBIM5 [22]	Kaimal 2017	Uganda	Pharmacy refill plus programme	Stable, not specified	624	08/2015 to 09/2016
FBIM6 [29]	Nsumba 2019	Uganda	Treatment failure management service	Unstable, not specified	862	03/2017
FBIM7 [30]	Mwila 2018	Zambia	FBO‐based community ART delivery	No restriction, not specified	5,014	12/2015 to 04/2018
**Out‐of‐facility‐based individual models (OFBIM)**						
OFBIM1 [31]	Tchissambou 2018	DRC	Community ART distribution centres	Stable, ≥15	2,027	01/2016 to 12/2017
OFBIM2 [25]	Kamerhe 2018	DRC	Community‐based point of ART distribution	Stable, ≥18	974	10/2016 to 03/2018
OFBIM3 [32]	Mothibi 2018	DRC	Community‐based individual drug distribution	Stable, ≥18	1,484	10/2016 to 09/2017
OFBIM4 [33]	Pasipamire 2019^b^	Eswatini	Comprehensive outreach	Stable, ≥16	918	02/2015 to 08/2016
OFBIM5 [34]	Avong 2018	Nigeria	Community pharmacy ART dispensing	Stable, ≥18	295	02/2016 to 05/2017
OFBIM6 [35]	Nwabueze 2018	Nigeria	Out‐of‐facility ARV delivery	Stable, not specified	283	03/2016 to 02/2017
OFBIM7 [24]	Fox 2019	South Africa	Decentralized medication delivery	Stable, ≥18	578	03/2016 to 09/2018
OFBIM8 [36]	Geldsetzer 2018	Tanzania	ARV community delivery	Stable, not specified	2,172	03/2016 to 10/2017
OFBIM9 [37]	Mulenga 2019	Zambia	Community pharmacy dispensing	Stable, not specified	237	08/2018 to 12/2018
OFBIM10 [38]	Mwanda 2018	Zambia	DSD model for prisoners	Stable, not specified	241	10/2016 to 03/2018
OFBIM11 [39]	Chibesakunda 2019	Zambia	Community ART centres	No restriction, not specified	7037	10/2016 to 01/2019
OFBIM12 [40]	Limbada 2019	Zambia	Home‐based delivery	Stable, ≥18	2,493	05/2017 to 12/2017
**Client‐led groups (CLG)**						
CLG1 [33]	Pasipamire 2019^b^	Eswatini	Community ART groups	Stable, ≥16	918	02/2015 to 08/2016
CLG2 [41]	Van Rompaey 2019	Mozambique	Community adherence groups	Stable, not specified	15,457	08/2016 to 12/2017
CLG3 [42]	Okechukwu 2018	Tanzania	Community ART refill groups	Stable, not specified	13,372	05/2017 to 11/2017
CLG4 [43]	Kagimu 2018	Uganda	Community client‐led ART delivery	Stable, not specified	14	10/2017–09/2018
CLG5 [44]	Mwamba 2018	Zambia	Community adherence groups and viral load clinic	Unstable, ≥14	386	10/2017 to 11/2018
**Healthcare worker‐led groups (HCWLG)**						
HCWLG1 [25]	Kamerhe 2018	DRC	ART support groups	No restriction, not specified	974	10/2016 to 03/2018
HCWLG2 [33]	Pasipamire 2019	Eswatini	Treatment clubs	Stable, ≥16	918	02/2015 to 08/2016
HCWLG3 [45]	Finci 2018	Mozambique	Adherence clubs	Stable, not specified	687	10/2015–03/2018
HCWLG4 [46]	Manganye 2018	South Africa	Adherence clubs	Stable, ≥18	141,269	10/2016 to 06/2018
HCWLG5 [47]	Meehan 2018	South Africa	Community‐based adherence clubs	Stable, ≥18	465	08/2017 to 11/2017
HCWLG6 [48]	Pahad 2018	South Africa	Youth care clubs	No restriction, 12 to 24	589	08/2016 to 12/2017
HCWLG7 [49]	Lebelo 2019^b^	South Africa	6 month‐refill adherence clubs	Stable, not specified	2,150	10/2017 to 02/2019
HCWLG8 [49]	Lebelo 2019^b^	South Africa	2 month‐refill adherence clubs	Stable, not specified	2,150	10/2017 to 02/2019
HCWLG9 [28]	Cassidy 2018	South Africa	Community clubs	Stable, not specified	4,753	10/2015 to 10/2017
HCWLG10 [28]	Cassidy 2018	South Africa	Facility clubs	Stable, not specified	4,753	10/2015 to 10/2017
HCWLG11 [23, 24]	Fox 2018; Fox 2019	South Africa	Adherence clubs	Stable, ≥18	569; 578	Pre‐/‐post 01/2015
HCWLG12 [50]	Roy 2018	Zambia	Urban adherence groups	Not specified, ≥14	1,096; 15 FGD	05/2016–10/2017
HCWLG13 [40]	Limbada 2019	Zambia	Adherence clubs	Stable, ≥18	5,252; 869	05/2017 to 12/2017

FBO, faith‐based organization; FGD, focus group discussion.

^a^ Most models where age was not specified appeared to be limited to adults

^b^ The authors used associated documents (e.g. published study protocols, unpublished reports) relevant to these source documents to supplement the DSD model description, if insufficient detail was provided in the publication itself

^c^ Sample sizes pertain to the entire study population rather than for a specific DSD model. For publications that evaluated different DSD models in each arm, we report the total N for the study cohort rather than the N in each study arm

^d^ For most models, stable was defined per national guidelines, though clinicians used clinical criteria to define stability when necessary laboratory tests were not available.

**Table 2:** Frequency of DSD model outcomes reported, by model category^a^

Outcome reported (n, %)	Facility‐based individual (FBIM) (n = 7)		Out‐of‐facility‐based individual (OFBIM) (n = 12)		Client‐led group (CLG) (n = 5)		Healthcare worker‐led group (HCWLG) (n = 13)		Total (n = 37)	
Retention	4	(57%)	10	(83%)	2	(40%)	13	(100%)	29	(78%)
Viral load suppression	6	(86%)	6	(50%)	3	(60%)	7	(54%)	22	(59%)
Adherence	0	(0%)	0	(0%)	1	(20%)	0	(0%)	1	(3%)
Prescription refill	0	(0%)	1	(8%)	1	(20%)	1	(8%)	3	(8%)

^a^ Most models reported more than outcome, resulting in column totals that are greater than the number of models in each category.

In addition to the models listed in Table 1, 11 source documents reported comparative results for a conventional care model, creating a total of 48 model‐instances with clinical outcomes included in this review (37 DSD + 11 conventional models). Out‐of‐facility‐based individual models (32%) and healthcare worker‐led group models (35%) were the most commonly reported categories (Table S5).

Three quarters (76%) of the models were limited to clinically stable patients, and most (59%) were for adults (Table 1). Definitions of stability varied. Some models required prior evidence of viral suppression, whereas others relied on clinical condition, for example and minimum duration on ART prior to model entry. Details of how a stable patient is defined are presented elsewhere [51].

Additional model characteristics are described in Table S6. Most models provided basic clinical care, antiretroviral medications (ARVs) and laboratory monitoring only (78%). Almost half (46%) included services delivered both in the clinic and in the community, rather than solely one or the other. For those that identified clinical care and pharmacy refill providers, nearly all clinical care (96%) was provided by trained clinicians, though few sources specified the clinical cadre involved; more than two‐thirds of medication refills (70%) were provided by non‐clinician staff (community health workers, designated patients or lay counsellors). More than half the models (57%) required patients to have a total of four to eight clinic visits or DSD model interactions per year; most of the rest required more than eight visits or interactions per year, though a few (18%) were structured for three or fewer per year (Table S6). Models that are focused on adolescents and children are often more intensive than those for adults, which could inflate the average frequency estimated here. As only one model in our review was aimed at adolescents, however, it is unlikely that this had a substantial impact on this estimate [48].

### 3.3 Outcomes

A total of 55 outcomes were reported for the 37 models included in the review (Table 2). Retention in care was the most common, reported for 78% of the models. Just over half the models (59%) reported viral suppression.

Quantitative results for each study are shown in Table 3. Some studies included effect sizes in comparison with conventional care, whereas others did not provide comparison values at all, but simply reported the outcomes of the DSD models. Table 4 provides additional information, including effect sizes, for studies that did report these measures. More detailed versions of both tables, including any estimates or calculations by the authors, can be found in Table S7.

**Table 3:** Clinical outcomes as reported for DSD and comparison models

Source	Model ID	Country	Model name	Timing of outcome	Differentiated model outcome (%)	Conventional care model outcome (%)	Difference (=DSD minus conventional)
Retention in care							
Facility‐based individual model							
Kamerhe 2018 [25]	FBIM1	DRC	Fast‐track ART refill	≤12 months	96.0%	60.0%	+33.7%
Cassidy 2018 [28]	FBIM4	South Africa	Quick pick‐up model	≤12 months	91.0%	86.0%	+5%
Kaimal 2018 [22]	FBIM5	Uganda	Pharmacy refill plus programme	≤12 months	99.3%		
Nsumba 2019 [29]	FBIM6	Uganda	Treatment failure management service	Unknown	47.3%		
Out‐of‐facility‐based individual model							
Kamerhe 2018 [25]	OFBIM2	DRC	Community‐based point of ART distribution	≤12 months	96.0%	60.0%	+33.7%
Mothibi 2018 [32]	OFBIM3	DRC	Community‐based individual drug distribution	≤12 months	96.5%		
Tchissambou 2018 [31]	OFBIM1	DRC	Community ART distribution centre	≤12 months	86.1%		
Pasipamire 2019 [33]	OFBIM4	Eswatini	Comprehensive outreach	≤12 months	90.8%		
Avong 2018 [34]	OFBIM5	Nigeria	Community pharmacy ART dispensing	≤12 months	97.2%^a^		
Nwabueze 2018 [35]	OFBIM6	Nigeria	Out‐of‐facility ART delivery	≤12 months	92.5%		
Fox 2019^b^ [24]	OFBIM7	South Africa	Decentralized medication delivery	≤12 months	81.5%	87.2%	−5.7%
Chibesakunda 2019 [39]	OFBIM11	Zambia	Community ART centres	≤12 months	83%		
Limbada 2019 [40]	OFBIM12	Zambia	Home‐based delivery	12 to 24 months	84.9%		
Mulenga 2019 [37]	OFBIM9	Zambia	Community pharmacy dispensation	≤12 months	100%		
Healthcare worker‐led group							
Kamerhe 2018 [25]	HCWLG1	DRC	ART support groups	≤12 months	93.7%	60.0%	+33.7%
Pasipamire 2019 [33]	HCWLG2	Eswatini	Comprehensive outreach	≤12 months	90.8%		
Finci 2018 [45]	HCWLG3	Mozambique	Adherence clubs	12 to 24 months	84.4%		
Cassidy 2018 [28]	HCWLG9	South Africa	Community clubs	≤12 months	89.9%	86.0%	+3.9%
Cassidy 2018 [28]	HCWLG10	South Africa	Facility clubs	≤12 months	85.1%	86.0%	+0.9%
Fox 2019^b^ [24]	HCWLG11	South Africa	Adherence clubs	≤12 months	89.5%	81.6%	+7.9%
Lebelo 2019^b^, ^c^ [49]	HCWLG7	South Africa	6 month‐refill adherence clubs	≤12 months	97%		
Lebelo 2019^b^, ^c^ [49]	HCWLG8		2 month‐refill adherence clubs	≤12 months	98%		
Manganye 2018 [46]	HCWLG4	South Africa	Adherence clubs	12 to 24 months	94.9%		
Meehan 2018 [47]	HCWLG5	South Africa	Community‐based adherence clubs	12 to 24 months	82.5%		
Pahad 2018 [48]	HCWLG6	South Africa	Youth care clubs	≤12 months	80.9%	84.0%	−3.1%
Limbada 2019 [40]	HCWLG13	Zambia	Adherence clubs	12 to 24 months	92.6%		
Roy 2018 [50]	HCWLG12	Zambia	Urban adherence groups	≤12 months	71.0%	42.0%	29.0%
Client‐led group							
Pasipamire 2019 [33]	CLG1	Eswatini	Community ART groups	≤12 months	94.4%		
Kagimu 2018 [43]	CLG4	Uganda	Community client‐led ART delivery	≤12 months	100.0%		
Viral load < 1000 copies/mm^3^							
*Facility‐based individual model*							
Havlir 2019 [26]	FBIM2	Kenya and Uganda	Patient‐centred streamlined care	Unknown	79%	68%	+9%
Mdala 2018 [27]	FBIM3	Namibia	Nurse‐initiated management of ART	Unknown	86.0%		
Cassidy 2018 [28]	FBIM4	South Africa	Quick pick‐up model	12 to 24 months	96.0%	91.0 %	+5%
Kaimal 2018 [22]	FBIM5	Uganda	Pharmacy refill plus programme	≤12 months	98.8%		
Nsumba 2019 [29]	FBIM6	Uganda	Treatment failure management service	Unknown	39.7%		
Mwila 2018 [30]	FBIM7	Zambia	FBO‐based community ART delivery	12 to 24 months	89.1%	83.8%^d^	+5.3%
Out‐of‐facility‐based individual model							
Mothibi 2018 [32]	OFBIM3	DRC	Community‐based individual drug distribution	≤12 months	98.5%		
Nwabueze 2018 [35]	OFBIM6	Nigeria	Out‐of‐facility ART delivery	≤12 months	100.0%		
Fox 2019^b^ [24]	OFBIM7	South Africa	Decentralized medication delivery	≤12 months	77.2%	74.3%	+2.9%
Geldsetzer 2018^b^ [36]	OFBIM8	Tanzania	ARV community delivery	≤12 months	90.3%	89.1%	+1.2%
Chibesakunda 2019 [39]	OFBIM11	Zambia	Community ART centres	≤12 months	90.4%	84.8%	+5.6%
Mwanda 2018 [38]	OFBIM10	Zambia	DSD model for prisoners	Unknown	91.7%		
Health care worker‐led group							
Finci 2018 [45]	HCWLG3	Mozambique	Adherence club	12 to 24 months	81.0%		
Cassidy 2018 [28]	HCWLG9	South Africa	Community clubs	12 to 24 months	98.0%	91.0%	+7.0%
Cassidy 2018 [28]	HCWLG10	South Africa	Facility clubs	12 to 24 months	94.9%	91.0%	+3.9%
Fox 2019^b^ [24]		South Africa	Adherence clubs	≤12 months	80.0%	79.6%	+0.4%
Lebelo 2019^b^, ^c^ [49]	HCWLG7	South Africa	6 month‐refill adherence clubs	≤12 months	97.8%		
Lebelo 2019^b^, ^c^ [49]	HCWLG8	South Africa	2 month‐refill adherence clubs	≤12 months	96.5%		
Pahad 2018 [48]	HCWLG6	South Africa	Youth care clubs	≤12 months	75.0%		
*Client‐led group*							
Kagimu 2018 [43]	CLG4	Uganda	Community client‐led ART delivery	≤12 months	100.0%		
Mwamba 2018 [44]	CLG5	Zambia	Community adherence groups and dedicated VL clinic for unstable patients	Unknown	27.8%		
Van Rompaey^e^ [41]	CLG2	Mozambique	Community adherence groups	Unknown	uOR = 1.16^e^		
Adherence							
Client‐led group							
Kagimu 2018 [43]	CLG4	Uganda	Community client‐led ART delivery	≤12 months	95.0%		
Prescription refill							
Avong 2018 [34]	OFBIM5	Nigeria	Community pharmacy ART dispensing	≤12 months	100.0%		
Fox 2018 [23]	HCWLG11	South Africa	Adherence clubs	≤12 months	92.0%	88.0%	+4%
Okechukwu 2018 [42]	CLG3	Tanzania	Community ART refill groups	≤12 months	97.9%	87.0%	+10.9%

^a^ Rate calculated by authors

^b^ Cluster randomized trial

^c^ The comparator is a 2‐month pick‐up model in adherence clubs which is counted as a DSD model, rather than conventional care. This source document was not reported as including the standard of care inTable S5

^d^ National average

^e^ Only the effect estimates were reported by the source.

**Table 4:** Clinical outcomes of DSD models with effect size estimates compared to conventional care

Source	Model ID	Country	Model Name	N	Outcome		Effect size		Outcome definition
					DSD model	Conventional model	Crude	Adjusted	
Retention									
Pasipamire 2019 [33]	OFBIM4	Eswatini	Comprehensive outreach	918	90.8	‐	HR = 0.68^a^ (0.30 to1.51)	HR = 0.58^a^ (0.25 to 1.35)	Patient loss to follow‐up (LTFU) was defined as not having a recorded visit for 120 days or more before database closure. LTFU from care was time from enrolment to the composite endpoint of LTFU and death, regardless whether the outcome occurred while enrolled in the care model or in routine facility‐based ART care. Retention was measured at 12 months
	CLG1		Community ART groups	918	94.4	‐	HR = 1.0 (reference)	HR = 1.0 (reference)	
	HCWLG2		Treatment clubs	918	94.4	‐	HR = 1.07^a^ (0.54 to 2.08)	HR = 1.09^a^ (0.54 to 2.22)	
Fox 2019^b^ [24]	OFBIM7	South Africa	Decentralized medication delivery	1,147	81.5	87.2	DID= −6.0% (−10.6% to − 1.5%)	DID=−5.9% (−12.5% to 0.8%)^c^	Retention in care at 12 months after eligibility defined as 100% ‐ % attrition, with attrition as the sum of reported deaths, loss to follow‐up and transfers. Loss to follow‐up was defined as failure to attend the clinic within 90 days of a scheduled appointment.
	HCWLG11		Adherence clubs	1,147	89.5	81.6	DID = 7.4% (2.9% to 11.9%)	DID = 8.3% (1.1% to 15.6%)^c^	
Roy 2018 [50]	HCWLG12	Zambia	Urban adherence groups	1,096	71.0	42.0	RR = 1.7 (1.32 to 2.22)^a^	‐	Cumulative incidence of the first late drug pick‐up at 12 months used as a proxy for retention^a^
Viral load < 1000 copies/mm^3^									
Havlir 2019^b^ [26]	FBIM2	Kenya and Uganda	Patient‐centred streamlined care	186,354	79	68	‐	PR (prevalence ratio) =1.15 (1.11 to 1.20)^d^	Viral suppression defined as HIV RNA < 500 copies per millilitre at 3 years (cross‐sectional measurement)
Van Rompaey [41]	CLG2	Mozambique	Community adherence groups	15,457	‐	‐	uOR = 1.16^e^		Viral suppression defined as HIV RNA < 75 copies per millilitre. Time of follow‐up remains unclear.
Fox 2019^b^ [24]	HCWLG11	South Africa	Adherence clubs	1,147	80	79.6	DID = 3.1% (−3.8% to 10.0%)	DID = 3.8% (−6.9% to 14.4%)^c^	Viral suppression (<400 copies/mL) at 12 months after eligibility
	OFBIM7		Decentralized medication delivery	1,266	77.2	74.3	DID= −0.5% (−7.5% to 6.6%)	DID= −1.0% (−12.2% to 10.1%)^c^	
Geldsetzer 2018^b^ [36]	OFBIM8	Tanzania	ARV community delivery	2,172	90.3	89.1	RR = 1.12 (0.80 to 1.59)^a^	RR = 1.00 (0.74 to 1.35)^a^, ^f^	Viral load ≥ 1000 copies/mL^a^ at 6 months
Prescription refill									
Fox 2018 [23]	HCWLG11	South Africa	Adherence clubs	579	92.0	88.0	DiD = 6.7% (3.4% to 10.4%)	DiD = 7.5% (−1.3% to 16.2%)^c^	Proportion of patients who picked up medication within four months of enrolling in the intervention.

^a^ Author calculated

^b^ Cluster randomized trial

^c^ Cluster and covariate adjusted

^d^ Adjusted for incomplete measures of HIV serostatus and HIV RNA with the use of individual‐level targeted maximum likelihood estimation (adjustment variables included sex, age group, marital status, educational level, occupation, alcohol use, household wealth, mobility, previous HIV testing and care status)

^e^ Only the effect estimates were reported

^f^ Adjusted for baseline viral load, CD4 count, age and sex.

### 3.4 Retention in care

Although retention in care was the most commonly reported outcome, only a few sources provided a comparison to conventional care. For those that did, retention in the DSD model was generally within 5% of that in conventional care, with the exception of a healthcare worker‐led group model in the Democratic Republic of Congo, which greatly improved retention [25]. Among those not providing a comparison, retention generally exceeded 80% (range 47% to 100%). For the few sources (n = 3) which reported retention outcomes with an effect size, effects varied widely, from much better than conventional care to somewhat worse.

### 3.5 Viral load suppression

Among the 22 models that reported viral load suppression, ten included a comparison with conventional care (including one that reported only an effect estimate and not actual values). All those with a comparison reported a small increase in suppression in the DSD model. Reported suppression exceeded 90% (range 77% to 98%) in 11/21 models. Five models reported viral suppression with an effect size estimate. Three of these found no difference in suppression when adjusting for baseline differences. Streamlined care in Uganda and Kenya [26] and CAGs in Mozambique [41] both reported approximately 15% (prevalence ratio = 1.15 and unadjusted odds ratio = 1.16 respectively) improvements in suppression.

### 3.6 Adherence and prescription refill rates

Few sources (n = 4) used adherence to ARVs or prescription refill rates as outcomes; results are shown in Table 3. Rates of adherence (n = 1) and prescription refill (n = 3) were >90% (range 92% to 100%) across the models. Only two reported a comparison with conventional care and the DSD model outperformed conventional care in both instances. No effect sizes were reported for adherence or prescription refill measures.

### 3.7 Quality of evidence

Among the three‐quarters of the sources included that were cohort studies and thus evaluated on the Newcastle‐Ottawa scale, the quality of the evidence was generally low to moderate (Table S8). Only two of the 22 cohort studies received a score of 7 points (high quality) on the 9‐point scale. The relatively low quality of evidence among cohort studies was due mainly to the absence of comparators in many of the studies and the scarcity of detail found in conference abstracts. Most of the remaining studies (n = 6) were randomized controlled trials, for which we assessed quality using the Cochrane Collaboration’s tool for assessing risk of bias cluster randomized trials (Table S9)[21]. All three full‐length articles (four models) were at low risk for bias [24, 26, 36] but a concern about bias applied to the two abstracts, driven mainly by the fact that the conference abstracts did not contain full information on study methodology [40, 49].

## 4 Discussion

We systematically reviewed and synthesized the current evidence related to clinical outcomes of differentiated service delivery models for HIV treatment in sub‐Saharan Africa between 2016 and 2019. While we identified 29 sources that described one or more clinical outcomes of 37 DSD models in 11 countries, only a minority (28%) compared the alternative models to conventional care or to one another, making it difficult to draw strong conclusions about the overall impact of DSD models on clinical outcomes. Because of the heterogeneity of outcome definitions and timing and the highly variable quality, size and scope of the studies included, we opted to present outcomes individually for each model, stratified by model category and outcome, rather than to estimate aggregate statistics.

For those models that did provide a comparison with conventional care, retention in care in DSD models was generally within 5% of that in conventional care, with a few exceptions that reported much better retention. Similarly, viral suppression was generally equivalent or slightly higher in the DSD models. We did not expect to see a marked improvement in clinical impact (retention or viral suppression) because most DSD models are limited to already‐stable patients, for whom outcomes can be sustained but cannot improve. Where comparisons with conventional care were provided and effect sizes reported, effects on retention and suppression varied widely, from slightly worse than conventional care to moderately better. In general, DSD models were not associated with a meaningful deterioration in patient outcomes, despite in many cases having fewer interactions with patients or relying on lower cadres of clinicians than did conventional care. These clinical indicators, while capturing the direct health benefit of DSD models, do not reflect patient experience of the model. The limited available qualitative data on patient satisfaction identified as part of the rapid systematic review have been reported elsewhere [52].

As is evident from the discussion earlier, this review had many limitations. While we believe that our search of the peer‐reviewed, published literature and abstracts was thorough, the lack of standard terminology for describing DSD models hampered the creation of precise search strings, and it is possible that some sources were missed. Most sources did not describe procedures for recruiting patients into DSD models, but it is possible that self‐ and provider‐selection biased participation towards the most motivated and empowered patients, among all those who met formal eligibility criteria. More important, the extreme heterogeneity of the sources that did meet inclusion criteria rendered any attempt to aggregate results or produce summary statistics misleading. This heterogeneity manifested itself in multiple ways. The topic of DSD models is highly diverse in itself. Evaluation methods ranged from single‐site, single‐arm observational cohorts to large randomized trials. The majority of sources did not provide comparisons with conventional care, and metrics for assessing outcomes varied widely and were in many cases poorly defined. The underlying patient populations were often poorly described, without disaggregation by age or sex, or were by design different by model even within countries. Finally, with the exception of the randomized trials that included a standard of care arm, outcomes reported reflect only what is happening with patients eligible for the DSD models, who in most cases were already stable on ART. By definition, these models increase the proportion of ineligible patients remaining in conventional care, whose outcomes may be worse. The outcomes reported for specific DSD models can thus not be regarded as overall ART programme outcomes.

Stemming from these limitations, the search reported here identifies gaps in the evidence base and research priorities for DSD model implementation in the coming years. In particular, rigorous evaluation of clinical outcomes, with relevant comparisons, is needed if we are to fully understand the implications of DSD models for HIV control. Longer term follow‐up under routine care settings, beyond the first 12 or 24 months, should be undertaken, as it is critical to know what happens to retention and viral suppression three, five, or ten years after entry into a DSD model. This is especially important when DSD models are focused on stable patients and large changes in treatment outcomes are unlikely in the short term. Evaluation reports on the outcomes of DSD models should consistently include a description of the population served, as models limited to already‐stable patients are likely to have different outcomes from those that enrol a cross section of the ART patient population. Wherever possible, evaluations should include an entire ART population (patients eligible for and not eligible for DSD models; patients enrolled or not enrolled in the models), so that overall treatment programme outcomes can be estimated, rather than only those for patients in the models. Finally, there is also a need for electronic medical record systems to evolve to capture data on DSD model participation, as this is an essential step towards understanding the true clinical and other impacts of DSD models.

## 5 Conclusions

We note that there is a difference between the clinical outcomes of the patient enrolled in DSD models and the “impact” of implementing DSD models as part of national HIV programmes. In many of the studies included in this review, only a small proportion of eligible patients were enrolled in a DSD model, and only those patients’ outcomes reported. The effect of those patients’ outcomes on the overall, aggregate outcomes of the healthcare facilities at which the DSD models were implemented may have been modest, or even trivial, if large numbers of other patients remained in conventional care. Future evaluations of the outcomes of DSD models would be of greater value if they considered the entire, relevant patient population—for example all the ART patients served by a facility, or all the ART patients in a catchment area—as the denominator for assessing success.

## Competing Interest

The authors declare that they have no conflicting interests.

## Authors’ Contributions

LL, SK and SR conceived of and designed the study. SK, SP, BEN, RC, CG and AH identified and reviewed sources and extracted data. LL, SK and SR analysed the data and drafted the manuscript. DF contributed to design and data collection. MF contributed to design and data analysis. All authors reviewed and edited the manuscript.

## Supporting information

Table S1. Inclusion/exclusion criteria for publications and abstracts
Table S2. Search strategy
Table S3. Reasons for exclusions after full‐text review
Table S4. Original source documents and models
Table S5. Characteristics of source documents
Table S6. Characteristics of service delivery models
Table S7. Details of treatment outcomes reported in the source documents
Table S8. Risk of bias assessment for cohort studies (Newcastle‐Ottawa Scale)
Table S9. Risk of bias assessment for cluster randomized trials (Cochrane Collaboration’s tool)
