# Supplementary material for: Retention in care and viral suppression in differentiated service delivery models for HIV treatment delivery in sub‐Saharan Africa: a rapid systematic review
Source: J Int AIDS Soc. 2020 Nov 28;23(11):e25640. doi: 10.1002/jia2.25640 (PMC7696000; doi:10.1002/jia2.25640)
Supplement: Supplementary file 1 — Table S1. Inclusion/exclusion criteria for publications and abstracts Table S2. Search strategy Table S3. Reasons for exclusions after full‐text review Table S4. Original source documents and models Table S5. Characteristics of source documents Table S6. Characteristics of service delivery models Table S7. Details of treatment outcomes reported in the source documents Table S8. Risk of bias assessment for cohort studies (Newcastle‐Ottawa Scale) Table S9. Risk of bias assessment for cluster randomized trials (Cochrane Collaboration’s tool) [file JIA2-23-e25640-s001.docx]

# **SUPPLEMENTARY TABLES**

#### **Supplementary Table 1. Inclusion/exclusion criteria for publications and abstracts**

| Parameter | Inclusion criteria | Exclusion criteria |
| --- | --- | --- |
| Population | All ages and sexes; confirmed HIV positive status; on any regimen of lifelong antiretroviral treatment | Pregnant women in PMTCT programs; on ART for HIV prevention (PEP or PrEP) |
| Geographic region | Sub-Saharan Africa | None |
| Intervention | Delivery of lifelong ART that differs from standard or conventional care in terms of at least one of population, location, frequency, or provider cadre | Report solely about standard or conventional model for delivering ART, absent any differentiation based on population, location, frequency, or provider cadre |
| Study design | Reports primary, patient-level data from retrospective or prospective cohorts collected under any study design (trial, observational) with or without a comparison group | Systematic or other reviews, case series or reports, treatment guidelines, mathematical models, editorials, commentaries |
| Required descriptive data about model | Describes all of patients, location, frequency, provider, provider cadre, and services provided (see below for further explanation of this criterion) | Insufficient description of all the characteristics needed to define the model |
| Comparator | Not required; single arm evaluations are eligible | None |
| Outcomes | Reports at least one of coverage of population in need, uptake of ART, clinical outcomes, costs/resource utilization, acceptability to patients or providers, or feasibility of implementation | Insufficient detail provided to estimate at least one outcome |
| Timing | A majority of follow up data report on the delivery of antiretroviral treatment on or after January 1, 2016 | A majority of data accrued before January 1, 2016 |
| Sector | Services provided to the public sector through government-managed public health infrastructure or through NGO/private programs or facilities that serve the uninsured sector | Services or programs for privately (commercially) insured patients |

### **Supplementary Table 2. Search strategy**

| **Web of science** | |
| --- | --- |
| #1 | TS=(Africa South of the Sahara OR Sub-Saharan Africa OR Subsaharan Africa OR Africa, Sub- Saharan) |
|  |  |
| #2 | \|  \| TS=(HIV Seropositiv* OR Seropositiv* HIV OR Seropositiv*, HIV OR AIDS Seropositiv* OR Seropositiv*, AIDS OR Anti-HIV Positiv* OR Anti HIV Positiv* OR HTLV-III Seropositiv* OR HTLV III Seropositiv* OR HIV Seroconversion* OR Seroconversion*, HIV OR HTLV-III Seroconversion* OR HTLV III Seroconversion* OR Seroconversion*, HTLV-III OR AIDS Seroconversion* OR Seroconversion*, AIDS OR HIV Antibody Positivity OR Antibody Positivities, HIV OR Antibody Positivity, HIV OR HIV Antibody Positivities OR Positivities, HIV Antibody OR Positivity, HIV Antibody OR HIV Infections OR HIV Infection OR Infection, HIV OR Infections, HIV OR HTLV-III-LAV Infections OR HTLV III LAV Infections OR HTLV-III-LAV Infection OR Infection, HTLV-III-LAV OR Infections, HTLV-III-LAV OR T-Lymphotropic Virus Type III Infections, Human OR T Lymphotropic Virus Type III Infections, Human OR HTLV-III Infections OR HTLV III Infections OR HTLV-III Infection OR Infection, HTLV-III OR Infections, HTLV-III OR HIV Coinfection OR Coinfection, HIV OR Coinfections, HIV OR HIV Coinfections) \| \| --- \| --- \| |
| #3 | TS=(Anti-HIV Agents OR Agents, Anti-HIV OR Anti HIV Agents OR Anti-AIDS Agents OR Agents, Anti-AIDS OR Anti AIDS Agents OR Anti-HIV Drugs OR Anti HIV Drugs OR Drugs, Anti-HIV OR AIDS Drugs OR Drugs, AIDS OR Anti-AIDS Drugs OR Anti AIDS Drugs OR Drugs, Anti-AIDS OR antiretroviral therap* OR Antiretroviral Therap*, Highly Active OR Highly Active Antiretroviral Therapy OR HAART) |
| #4 | \|  \| #3 AND #2 AND #1 \| \| --- \| --- \| |
| #5 | \|  \| TS=(Drug Evaluation OR Drug Evaluations OR Evaluation, Drug OR Evaluations, Drug OR Evaluation Studies, Drug OR Drug Evaluation Studies OR Drug Evaluation Study OR Evaluation Study, Drug OR Studies, Drug Evaluation OR Study, Drug Evaluation OR Drug Approval OR Approval, Drug OR Approvals, Drug OR Drug Approvals OR Food and Drug Administration Drug Approval OR Drug Approval Process OR Approval Process, Drug OR Approval Processes, Drug OR Drug Approval Processes OR Process, Drug Approval OR Processes, Drug Approval OR New Drug Approval Process OR New Drug Approval OR Approval, New Drug OR Approvals, New Drug OR Drug Approval, New OR Drug Approvals, New OR New Drug Approvals OR Food and Drug Administration Drug Approval Process OR "Clinical Trials" OR "Clinical Trial") \| \| --- \| --- \| |
| #6 | #4 NOT #5 |
| #7 | #4 NOT #5 Timespan=2016-2018 |
| **Embase** | |
| #1 | 'africa south of the sahara'/exp OR 'africa south of the sahara' OR 'black africa' OR 'sub saharan africa' OR 'subsaharan africa' |
| #2 | 'human immunodeficiency virus infection'/exp OR 'hiv infection' OR 'hiv infections' OR 'human immunodeficiency virus infection' OR 'hiv seropositivity' OR 'human immunodeficiency virus encephalopathy' |
| #3 | 'anti human immunodeficiency virus agent'/exp OR 'anti hiv agent' OR 'anti hiv agents' OR 'anti human immunodeficiency virus agent' OR 'anti-hiv agents' OR 'highly active antiretroviral therapy'/exp OR 'haart' OR 'antiretroviral therapy, highly active' OR 'highly active antiretroviral theraphy' |
| #4 | 'drug screening'/exp OR 'assay, subrenal capsule' OR 'drug evaluation' OR 'drug scanning' OR 'drug screening' OR 'drug screening assays, antitumor' OR 'drug screening assays, antitumour' OR 'drug testing' OR 'drug trial' OR 'pharmaceutical screening' OR 'screening, drug' OR 'testing, drug' OR 'tumour stem cell assay' OR 'xenograft model antitumor assays' OR 'xenograft model antitumour assays' |
| #5 | #1 AND #2 AND #3 |
| #6 | #4 NOT #5 |
| #7 | #5 NOT #4 |
| #8 | #5 NOT #4 AND [2016-2018]/py |
| **Pubmed** | |
|  | (((("Africa South of the Sahara"[Mesh] OR Sub-Saharan Africa OR Subsaharan Africa OR Africa, Sub-Saharan)) AND ((((("HIV Seropositivity"[Mesh] OR HIV Seropositivities OR Seropositivities, HIV OR Seropositivity, HIV OR AIDS Seropositivity OR AIDS Seropositivities OR Seropositivities, AIDS OR Seropositivity, AIDS OR Anti-HIV Positivity OR Anti HIV Positivity OR Anti-HIV Positivities OR Positivities, Anti-HIV OR Positivity, Anti-HIV OR HTLV-III Seropositivity OR HTLV III Seropositivity OR HTLV-III Seropositivities OR Seropositivities, HTLV-III OR Seropositivity, HTLV-III OR HIV Seroconversion OR HIV Seroconversions OR Seroconversion, HIV OR Seroconversions, HIV OR HTLV-III Seroconversion OR HTLV III Seroconversion OR HTLV-III Seroconversions OR Seroconversion, HTLV-III OR Seroconversions, HTLV-III OR AIDS Seroconversion OR AIDS Seroconversions OR Seroconversion, AIDS OR Seroconversions, AIDS OR HIV Antibody Positivity OR Antibody Positivities, HIV OR Antibody Positivity, HIV OR HIV Antibody Positivities OR Positivities, HIV Antibody OR Positivity, HIV Antibody)) OR ("HIV Infections" [Mesh] OR HIV Infection OR Infection, HIV OR Infections, HIV OR HTLV-III-LAV Infections OR HTLV III LAV Infections OR HTLV-III-LAV Infection OR Infection, HTLV-III-LAV OR Infections, HTLV-III-LAV OR T-Lymphotropic Virus Type III Infections, Human OR T Lymphotropic Virus Type III Infections, Human OR HTLV-III Infections OR HTLV III Infections OR HTLV-III Infection OR Infection, HTLV-III OR Infections, HTLV-III OR HIV Coinfection OR Coinfection, HIV OR Coinfections, HIV OR HIV Coinfections))) AND ((("Anti-HIV Agents"[Mesh] OR Agents, Anti-HIV OR Anti-HIV Agents OR Anti-AIDS Agents OR Agents, Anti-AIDS OR Anti AIDS Agents OR Anti-HIV Drugs OR Anti HIV Drugs OR Drugs, Anti-HIV OR AIDS Drugs OR Drugs, AIDS OR Anti-AIDS Drugs OR Anti AIDS Drugs OR Drugs, Anti-AIDS OR "antiretroviral therapy"[TIAB])) OR ("Antiretroviral Therapy, Highly Active"[Mesh] OR Highly Active Antiretroviral Therapy OR HAART))))) NOT (((("Drug Evaluation"[Mesh] OR Drug Evaluations OR Evaluation, Drug OR Evaluations, Drug OR Evaluation Studies, Drug OR Drug Evaluation Studies OR Drug Evaluation Study OR Evaluation Study, Drug OR Studies, Drug Evaluation OR Study, Drug Evaluation)) OR ("Drug Approval"[Mesh] OR Approval, Drug OR Approvals, Drug OR Drug Approvals OR Food and Drug Administration Drug Approval OR Drug Approval Process OR Approval Process, Drug OR Approval Processes, Drug OR Drug Approval Processes OR Process, Drug Approval OR Processes, Drug Approval OR New Drug Approval Process OR New Drug Approval OR Approval, New Drug OR Approvals, New Drug OR Drug Approval, New OR Drug Approvals, New OR New Drug Approvals OR Food and Drug Administration Drug Approval Process)) OR ("Clinical Trials as Topic"[Mesh] OR Clinical Trial as Topic |

**Conferences for which abstracts were searched**

- International AIDS Society (IAS) 2016, 2017, 2018, 2019
- Conference on Retroviruses and Opportunistic Infections (CROI) 2016, 2017, 2018, 2019
- South African AIDS Conference (SAAIDS) 2017
- Southern African HIV Clinicians Society (SAHIVSOC) 2016, 2018
- European AIDS Conference (EACS) 2017
- INTEREST Conference 2016, 2017, 2018
- Zambia Health Research Conference (ZHRC) 2018
- Asia Pacific AIDS & Co-infections Conference (APACC) 2016, 2017
- International Conference on AIDS and STIs in Africa (ICASA) 2017

### **Supplementary Table 3. Reasons for exclusions after full-text review**

| **Reason for exclusion, n (%)** | **Manuscripts (n=94)** | | **Abstracts (n=105)** | | **Total (n=199)** | |
| --- | --- | --- | --- | --- | --- | --- |
| Study time period (majority of data from before 2016) | 55 | (60%) | 18 | (20%) | 73 | (40%) |
| Incomplete model description | 2 | (2%) | 30 | (33%) | 32 | (18%) |
| Study population type | 4 | (4%) | 24 | (27%) | 28 | (15%) |
| Study design | 11 | (12%) | 3 | (3%) | 14 | (8%) |
| Not a DSD model | 11 | (12%) | 3 | (3%) | 14 | (8%) |
| Publication type | 7 | (8%) | 0 | (0%) | 7 | (4%) |
| Study setting | 0 | (0%) | 6 | (7%) | 6 | (3%) |
| Abstract already published as full manuscript | 0 | (0%) | 4 | (4%) | 4 | (2%) |
| Duplicate data | 0 | (0%) | 2 | (2%) | 2 | (1%) |
| Study retracted | 1 | (1%) | 0 | (0%) | 1 | (1%) |
| No clinical outcome reported | 3 | (3%) | 15 | (14%) | 18 | (9%) |

### **Supplementary Text 1. PRISMA checklist**

| **Section and topic** | **Item No** | **Checklist item** | **(Page No.#)** |
| --- | --- | --- | --- |
| **ADMINISTRATIVE INFORMATION** | | |  |
| Title: |  |  |  |
| Identification | 1a | Identify the report as a protocol of a systematic review | 1 |
| Update | 1b | If the protocol is for an update of a previous systematic review, identify as such | 1 |
| Registration | 2 | If registered, provide the name of the registry (such as PROSPERO) and registration number | 2 |
| Authors: |  |  |  |
| Contact | 3a | Provide name, institutional affiliation, e-mail address of all protocol authors; provide physical mailing address of corresponding author | 1 |
| Contributions | 3b | Describe contributions of protocol authors and identify the guarantor of the review | 8 |
| Amendments | 4 | If the protocol represents an amendment of a previously completed or published protocol, identify as such and list changes; otherwise, state plan for documenting important protocol amendments | 7 |
| Support: |  |  |  |
| Sources | 5a | Indicate sources of financial or other support for the review | 8 |
| Sponsor | 5b | Provide name for the review funder and/or sponsor |  |
| Role of sponsor or funder | 5c | Describe roles of funder(s), sponsor(s), and/or institution(s), if any, in developing the protocol |  |
| **INTRODUCTION** | | |  |
| Rationale | 6 | Describe the rationale for the review in the context of what is already known | 3 |
| Objectives | 7 | Provide an explicit statement of the question(s) the review will address with reference to participants, interventions, comparators, and outcomes (PICO) | 3-4 |
| **METHODS** | | |  |
| Eligibility criteria | 8 | Specify the study characteristics (such as PICO, study design, setting, time frame) and report characteristics (such as years considered, language, publication status) to be used as criteria for eligibility for the review | 4-5 |
| Information sources | 9 | Describe all intended information sources (such as electronic databases, contact with study authors, trial registers or other grey literature sources) with planned dates of coverage | 5 |
| Search strategy | 10 | Present draft of search strategy to be used for at least one electronic database, including planned limits, such that it could be repeated | 5 and Appendix-A |
| Study records: |  |  |  |
| Data management | 11a | Describe the mechanism(s) that will be used to manage records and data throughout the review | 6 |
| Selection process | 11b | State the process that will be used for selecting studies (such as two independent reviewers) through each phase of the review (that is, screening, eligibility and inclusion in meta-analysis) | 6 |
| Data collection process | 11c | Describe planned method of extracting data from reports (such as piloting forms, done independently, in duplicate), any processes for obtaining and confirming data from investigators | 7 |
| Data items | 12 | List and define all variables for which data will be sought (such as PICO items, funding sources), any pre-planned data assumptions and simplifications | 7 |
| Outcomes and prioritization | 13 | List and define all outcomes for which data will be sought, including prioritization of main and additional outcomes, with rationale | 4 |
| Risk of bias in individual studies | 14 | Describe anticipated methods for assessing risk of bias of individual studies, including whether this will be done at the outcome or study level, or both; state how this information will be used in data synthesis | 7 |
| Data synthesis | 15a | Describe criteria under which study data will be quantitatively synthesized | 7 |
|  | 15b | If data are appropriate for quantitative synthesis, describe planned summary measures, methods of handling data and methods of combining data from studies, including any planned exploration of consistency (such as I^2^, Kendall’s τ) |  |
|  | 15c | Describe any proposed additional analyses (such as sensitivity or subgroup analyses, meta-regression) |  |
|  | 15d | If quantitative synthesis is not appropriate, describe the type of summary planned |  |
| Meta-bias(es) | 16 | Specify any planned assessment of meta-bias(es) (such as publication bias across studies, selective reporting within studies) | 7 |
| Confidence in cumulative evidence | 17 | Describe how the strength of the body of evidence will be assessed (such as GRADE) | 7 |

* The page numbers in this table reference the systematic review protocol published at [PROSPERO CRD42019118230](https://www.crd.york.ac.uk/PROSPERO/display_record.php?ID=CRD42019118230&ID=CRD42019118230)

### **Supplementary Table 4. Original source documents and models**

| **Model codes** | **Source** | **Country** | **Models reported** | **Study Design** | **Follow-up period** | **Cohort Size** | **Population eligible** |
| --- | --- | --- | --- | --- | --- | --- | --- |
| OFBIM5 | Avong 2018 | Nigeria | Community pharmacy ART dispensing | Cohort | 12 | 295 | Stable,≥18 |
| HCWLG6 | Pahad 2018 | South Africa | Youth care clubs | Cohort | 12 | 589 | No restriction, 12-24 |
| HCWLG9 HCWLG10  FBIM4 | Cassidy 2018 | South Africa | Quick pick-up model (QPUP); Community club; Facility club | Cohort | 12 | 4,753 | Stable, not specified |
| OFBIM11 | Chibesakunda 2019 | Zambia | Community ART centres | Cohort | 28 | 7037 | No restriction, not specified |
| HCWLG3 | Finci 2018 | Mozambique | Adherence clubs | Cohort | 24 | 687 | Stable, not specified |
| HCWLG11 | Fox 2018 | South Africa | Adherence clubs | RCT | 4 | 569 | Stable,≥18 |
| HCWLG11  OFBIM7 | Fox 2019 | South Africa | Adherence clubs and decentralized medication delivery | RCT | 18 | 1,147 | Stable,≥18 |
| OFBIM8 | Geldsetzer 2018 | Tanzania | ARV community delivery | RCT | 17 | 2,172 | Stable, not specified |
| FBIM2 | Havlir 2019 | Kenya and Uganda | Patient-centered streamlined care | RCT | 36 | 186,354 | No restriction, not specified |
| CLG4 | Kagimu 2018 | Uganda | Community client led ART delivery (CCLAD) | Cohort | 12 | 14 | Stable, not specified |
| FBIM5 | Kaimal 2017 | Uganda | Pharmacy refill plus program (PRPP) | Cross-sectional | NA | 624 | Stable, not specified |
| OFBIM2  FBIM1  HCWLG1 | Kamerhe 2018 | DRC | ART support group; Community-based point of ART distribution (PoDi+); Fast-track ART refill circuit | Cohort | 12 | 974 | Stable,≥18 |
| HCWLG7  HCWLG8 | Lebelo 2018 | South Africa | 6 month-refill adherence clubs; 2 month-refill adherence clubs | RCT | 28 | 2,150 | Stable, not specified |
| OFBIM12  HCWLG13 | Limbada 2019 | Zambia | Home-based delivery; Adherence clubs | RCT | 18 | 2,493 | Stable,≥18 |
| HCWLG4 | Manganye 2018 | South Africa | Adherence club | Cohort | 18 | 141,269 | Stable,≥18 |
| FBIM3 | Mdala 2018 | Namibia | Nurse-initiated management of ART | Cohort | 24 | 18,367 | No restriction, not specified |
| HCWLG5 | Meehan 2018 | South Africa | Community based adherence club | Cohort | 24 | 465 | Stable,≥18 |
| OFBIM3 | Mothibi 2018 | DRC | Community-based individual drug distribution (PODI) | Cohort | 10 | 1,484 | Stable,≥18 |
| OFBIM9 | Mulenga 2019 | Zambia | Community pharmacy dispensation | Cohort | 6 | 237 | Stable, not specified |
| CLG5 | Mwamba 2018 | Zambia | Community adherence groups (CAG) & VL clinic | Cohort | 12 | 386 | Unstable, ≥14 |
| OFBIM10 | Mwanda 2018 | Zambia | DSD model for prisoners | Cohort | 48 | 241 | Stable, not specified |
| FBIM7 | Mwila 2018 | Zambia | FBO based community ART delivery | Cohort | 24 | 5,014 | No restriction, not specified |
| FBIM6 | Nsumba 2019 | Uganda | Treatment failure management service | Cohort | NA | 862 | Unstable, not specified |
| OFBIM6 | Nwabueze 2018 | Nigeria | Out-of-facility ARV delivery | Cohort | 12 | 283 | Stable, not specified |
| CLG3 | Okechukwu 2018 | Tanzania | Community ART refill groups | Cohort | 6 | 13,372 | Stable, not specified |
| HCWLG2  CLG1  HCWLG2 | Pasipamire 2018 | Swaziland | Community ART groups; Comprehensive outreach; Treatment clubs | Cohort | 18 | 918 | Stable, ≥16 |
| HCWLG12 | Roy 2018 | Zambia | Urban adherence groups (UAG) | Cohort | 12 | 1,096 | Not specified, ≥14 |
| OFBIM1 | Tchissambou 2018 | DRC | Community ART distribution center (PODI) | Cohort | 12 | 2,027 | Stable, ≥15 |
| CLG2 | Van Rompaey 2019 | Mozambique | Community adherence groups | Cohort | NA | 15,457 | Stable, not specified |

### **Supplementary Table 5. Characteristics of source documents**

| **Characteristic** | **Articles** | | **Abstracts** | | **Total** | |
| --- | --- | --- | --- | --- | --- | --- |
| **By source documents** |  |  |  |  |  |  |
| Total source documents included | 5 | (100%) | 24 | (100%) | 29 | (100%) |
| Reported conventional care comparison, in addition to DSD model | 3 | (60%) | 7 | (29%) | 10 | (34%) |
| Study type |  | |  | |  | |
| Cohort | 2 | (40%) | 20 | (83%) | 22 | (76%) |
| Randomized control trial | 3 | (60%) | 3 | (13%) | 6 | (21%) |
| Cross-sectional | 0 | (0%) | 1 | (4%) | 1 | (3%) |
| **By models** |  |  |  |  |  |  |
| Total DSD models included (excludes conventional care) | 8 | (100%) | 29 | (100%) | 37 | (100%) |
| Study location* |  |  |  |  |  |  |
| DRC | 0 | (0%) | 5 | (17%) | 5 | (14%) |
| Kenya | 1 | (13%) | 0 | (0%) | 1 | (3%) |
| Mozambique | 0 | (0%) | 2 | (7%) | 2 | (5%) |
| Namibia | 0 | (0%) | 1 | (3%) | 1 | (3%) |
| Nigeria | 1 | (13%) | 1 | (3%) | 2 | (5%) |
| South Africa | 2 | (25%) | 8 | (28%) | 10 | (27%) |
| Swaziland | 3 | (38%) | 0 | (0%) | 3 | (8%) |
| Tanzania | 1 | (13%) | 1 | (3%) | 2 | (5%) |
| Uganda | 1 | (13%) | 3 | (10%) | 4 | (11%) |
| Zambia | 0 | (0%) | 8 | (28%) | 8 | (22%) |
| Model classification |  |  |  |  |  |  |
| Facility based individual (FBIM) | 1 | (13%) | 6 | (21%) | 7 | (19%) |
| Out of facility based individual (OFBIM) | 4 | (50%) | 8 | (28%) | 12 | (32%) |
| Client led groups (CLG) | 1 | (13%) | 4 | (14%) | 5 | (14%) |
| Healthcare worker led groups (HCWLG) | 2 | (25%) | 11 | (38%) | 13 | (35%) |

*One study reported the same model in both Kenya and Uganda and was thus counted twice.

### **Supplementary Table 6. Characteristics of service delivery models**

| **Characteristic (n, %)** | **Facility based individual (FBIM) (N=7)** | | **Out of facility individual**  **(OFBIM)**  **(N=12)** | | **Client led group**  **(CLG)**  **(N=5)** | | **Healthcare worker led group**  **(HCWLG)**  **(N=13)** | | **Total**  **(N=37)** | |
| --- | --- | --- | --- | --- | --- | --- | --- | --- | --- | --- |
| **Provider** |  | |  | |  | |  | |  | |
| Clinical care provider |  |  |  |  |  |  |  |  |  |  |
| Medical doctor/medical officer | 2 | (29%) | 1 | (8%) | 0 | (0%) | 0 | (0%) | 3 | (8%) |
| Nurse | 3 | (43%) | 1 | (8%) | 0 | (0%) | 2 | (15%) | 6 | (16%) |
| Community health worker | 0 | (0%) | 0 | (0%) | 0 | (0%) | 1 | (8%) | 1 | (3%) |
| Non-specified clinician | 1 | (14%) | 5 | (42%) | 1 | (20%) | 8 | (62%) | 15 | (41%) |
| Unclear/not reported | 1 | (14%) | 5 | (42%) | 4 | (80%) | 2 | (15%) | 12 | (32%) |
| ART dispenser |  |  |  |  |  |  |  |  |  |  |
| Pharmacist | 3 | (43%) | 2 | (17%) | 0 | (0%) | 1 | (8%) | 6 | (16%) |
| Nurse | 3 | (43%) | 0 | (0%) | 0 | (0%) | 0 | (0%) | 3 | (8%) |
| Community health worker | 0 | (0%) | 3 | (25%) | 0 | (0%) | 3 | (23%) | 6 | (16%) |
| Designated patient | 0 | (0%) | 0 | (0%) | 2 | (40%) | 2 | (15%) | 4 | (11%) |
| Lay counselor | 0 | (0%) | 4 | (33%) | 1 | (20%) | 6 | (46%) | 11 | (30%) |
| Unclear/not reported | 1 | (14%) | 3 | (25%) | 2 | (40%) | 1 | (8%) | 7 | (19%) |
| **Location** |  |  |  |  |  |  |  |  |  |  |
| All clinic-based | 7 | (100%) | 0 | (0%) | 1 | (20%) | 7 | (54%) | 15 | (41%) |
| Mixed | 0 | (0%) | 7 | (58%) | 4 | (80%) | 6 | (46%) | 17 | (46%) |
| All community-based | 0 | (0%) | 5 | (42%) | 0 | (0%) | 0 | (0%) | 5 | (14%) |
| **Services provided** |  |  |  |  |  |  |  |  |  |  |
| C/A/L* | 5 | (71%) | 10 | (83%) | 4 | (80%) | 10 | (77%) | 29 | (78%) |
| C/A/L + health education | 0 | (0%) | 0 | (0%) | 0 | (0%) | 1 | (8%) | 1 | (3%) |
| C/A/L + index HIV testing | 0 | (0%) | 1 | (8%) | 0 | (0%) | 0 | (0%) | 1 | (3%) |
| C/A/L + reproductive counseling | 0 | (0%) | 0 | (0%) | 1 | (20%) | 2 | (15%) | 3 | (8%) |
| C/A/L + NCD care | 1 | (14%) | 0 | (0%) | 0 | (0%) | 0 | (0%) | 1 | (3%) |
| C/A/L + antenatal and childcare | 0 | (0%) | 1 | (8%) | 0 | (0%) | 0 | (0%) | 1 | (3%) |
| Not reported | 1 | (14%) | 0 | (0%) | 0 | (0%) | 0 | (0%) | 1 | (3%) |
| **Frequency of healthcare system interactions/year** |  |  |  |  |  |  |  |  |  |  |
| ≤3 | 1 | (14%) | 1 | (8%) | 0 | (0%) | 3 | (23%) | 5 | (14%) |
| 4-8 | 3 | (43%) | 7 | (58%) | 0 | (0%) | 6 | (46%) | 16 | (43%) |
| 8-12 | 0 | (0%) | 1 | (8%) | 1 | (20%) | 3 | (23%) | 5 | (14%) |
| >12 | 0 | (0%) | 1 | (8%) | 1 | (20%) | 0 | (0%) | 2 | (5%) |
| Not reported | 3 | (43%) | 2 | (17%) | 3 | (60%) | 1 | (8%) | 9 | (24%) |

***C/A/L = Basic clinical care, ARVs, and laboratory monitoring

### **Supplementary Table 7. Details of treatment outcomes reported in the source documents**

| **Source** | **Model ID** | **Country** | **Model name** | **DSD** | | | **Conventional care** |
| --- | --- | --- | --- | --- | --- | --- | --- |
|  |  |  |  | **Timing unknown or cross-sectional** | **≤ 12 months** | **12-24 months** |  |
| **RETENTION (%)** |  |  |  |  |  |  |  |
| **Facility based individual model** | |  |  |  |  |  |  |
| Kamerhe 2018 | FBIM1 | DRC | Fast-track ART refill |  | 96.0 (312/325)^a^ |  | 60.0 (NA) |
| Cassidy 2018 | FBIM4 | South Africa | Quick pick-up model |  | 91.0 (224/246)^a^ |  | 86.0 (731/850)^a^ |
| Kaimal 2018 | FBIM5 | Uganda | Pharmacy refill plus program |  | 99.3 (286/288)^a^ |  |  |
| Nsumba 2019 | FBIM6 | Uganda | Treatment failure management service | 47.3 (106/224) |  |  |  |
| **Out of facility based individual model** | |  |  |  |  |  |  |
| Mothibi 2018 | OFBIM3 | DRC | Community-based individual drug distribution |  | 96.5 (336/348)^c^ |  |  |
| Tchissambou 2018 | OFBIM1 | DRC | Community ART distribution center |  | 86.1 (1,745/2,027) |  |  |
| Kamerhe 2018 | OFBIM2 | DRC | Community-based point of ART distribution |  | 96.0 (577/601)^a^ |  | 60.0 (NA) |
| Pasipamire 2019 | OFBIM4 | Eswatini | Comprehensive outreach |  | 98.0 (88/98)^a^ |  |  |
| Avong 2018 | OFBIM5 | Nigeria | Community pharmacy ART dispensing |  | 97.2 (287/295) ^b^ |  |  |
| Nwabueze 2018 | OFBIM6 | Nigeria | Out-of-facility ART Delivery |  | 92.5 (262/283) |  |  |
| Fox 2019 | OFBIM7 | South Africa | Decentralized medication delivery |  | 81.5 (189/232)^a^ |  | 87 (301/345)^a^ |
| Mulenga 2019 | OFBIM9 | Zambia | Community  pharmacy dispensation |  | 100 (237/237) |  |  |
| Chibesakunda 2018 | OFBIM11 | Zambia | Community ART centers |  | 83 (NA) |  |  |
| Limbada 2019 | OFBIM12 | Zambia | Home-based delivery |  |  | 84.9 (715/842) |  |
| **Healthcare worker led group** | |  |  |  |  |  |  |
| Kamerhe 2018 | HCWLG1 | DRC | ART support group |  | 93.7 (45/48)^a^ |  | 60.0 (NA) |
| Pasipamire 2019 | HCWLG2 | Eswatini | Treatment clubs |  | 94.4 (273/289)^a^ |  |  |
| Finci 2018 | HCWLG3 | Mozambique | Adherence club |  |  | 84.4 (578/685)^e^ |  |
| Cassidy 2018^ⱡ^ | HCWLG9 | South Africa | Community club |  | 89.9 (402/447) |  | 86.0  (731/850)^a^ |
| Cassidy 2018^ⱡ^ | HCWLG10 | South Africa | Facility club |  | 85.1 (114/134) |  | 86.0  (731/850)^a^ |
| Meehan 2018a | HCWLG5 | South Africa | Community based adherence club |  |  | 82.5 (66/80)^a^ |  |
| Pahad 2018 | HCWLG6 | South Africa | Youth care clubs |  | 80.9 (477/589)^a^ |  | 84.0 (NA) |
| Manganye 2018 | HCWLG4 | South Africa | Adherence club |  |  | 94.9 (134,205/141,269) |  |
| Fox 2019 | HCWLG11 | South Africa | Adherence clubs |  | 89.5 (246/275)^a^ |  | 81.6 (196/240)^a^ |
| Limbada 2019 | HCWLG13 | Zambia | Adherence  clubs |  |  | 92.6 (743/802) |  |
| Roy 2018 | HCWLG12 | Zambia | Urban adherence groups |  | 71.0 (422/594)^a^ |  | 42.0 (211/502)^a^ |
| Lebelo 2019^§^ | HCWLG7 | South Africa | 6 month-refill adherence clubs |  | 97.0 (NA) |  |  |
| Lebelo 2019 | HCWLG8 | South Africa | 2 month-refill adherence clubs |  | 98.0 (NA) |  |  |
| **Client led group** |  |  |  |  |  |  |  |
| Pasipamire 2019 | CLG1 | Eswatini | Community ART groups |  | 94.4 (501/532)^a^ |  |  |
| Kagimu 2018 | CLG4 | Uganda | Community client led ART delivery |  | 100.0 (14/14) |  |  |
| **VL<1000 (%)** |  |  |  |  |  |  |  |
| **Facility based individual model** | |  |  |  |  |  |  |
| Havlir 2019 | FBIM2 | Kenya and Uganda | Patient-centered streamlined care | 79.0 (5,372/6,800)^a^ |  |  | 68.0 (4115/6,051)^a^ |
| Mdala 2018 | FBIM3 | Namibia | Nurse-initiated management of ART | 86.0 (15,796/18,367)^c^ |  |  |  |
| Cassidy 2018^ⱡ^ | FBIM4 | South Africa | Quick pick-up model |  |  | 96.0 (579/603)^a^ | 91.0 (1,369/1,504)^a^ |
| Kaimal 2018 | FBIM5 | Uganda | Pharmacy refill plus program |  | 98.8 (83/84) |  |  |
| Nsumba 2019 | FBIM6 | Uganda | Treatment failure management service | 39.7 (89/224) |  |  |  |
| Mwila 2018 | FBIM7 | Zambia | FBO based community ART delivery |  |  | 89.1  (4,472/5,014)^a^ | 83.8  (NA)^d^ |
| **Out of facility based individual model** | |  |  |  |  |  |  |
| Mothibi 2018 | OFBIM9 | DRC | Community-based individual drug distribution |  | 98.5  (1,025/1,040)^c^ |  |  |
| Nwabueze 2018 | OFBIM13 | Nigeria | Out-of-facility ART delivery |  | 100.0 (283/283) |  |  |
| Fox 2019 | OFBIM5 | South Africa | Decentralized medication delivery |  | 77.2 (179/232^)a^ |  | 74.3  (1901/257)^a^ |
| Geldsetzer 2018 | OFBIM6 | Tanzania | ARV community delivery |  | 90.3 (852/943)^a^ |  | 89.1 (777/872)^a^ |
| Mwanda 2018 | OFBIM11 | Zambia | DSD model for prisoners | 91.7  (221/241) |  |  |  |
| Chibesakunda 2019 | OFBIM2 | Zambia | Community ART centres |  | 90.4 (NA) |  | 84.8 (NA) |
| **Health care worker led group** | |  | |  |  |  |  |
| Finci 2018 | HCWLG3 | Mozambique | Adherence club |  |  | 81.0 (543/670)^e^ |  |
| Pahad 2018 | HCWLG6 | South Africa | Youth care clubs |  | 75.0 (442/589)^a^ |  |  |
| Cassidy 2018^ⱡ^ | HCWLG9 | South Africa | Community club |  |  | 98.0 (1,091/1,113)^a^ | 91.0  (1,369/1,504)^a^ |
| Cassidy 2018^ⱡ^ | HCWLG10 | South Africa | Facility club |  |  | 94.9 (357/376)^a^ | 91.0  (1,369/1,504)^a^ |
| Lebelo 2019^¥§^ | HCWLG7 | South Africa | 6 month-refill adherence clubs |  | 97.8 (NA) |  |  |
| Lebelo 2019 | HCWLG8 | South Africa | 2 month-refill adherence clubs |  | 96.5(NA) |  |  |
| Fox 2019 | HCWLG11 | South Africa | Adherence clubs |  | 80.0 (220/275)^a^ |  | 79.6  (234/294)^a^ |
| **Client led group** |  |  |  |  |  |  |  |
| Van Rompaey | CLG2 | Mozambique | Community adherence groups | uOR=1.16 |  |  |  |
| Kagimu 2018 | CLG4 | Uganda | Community client led ART delivery |  | 100.0 (14/14) |  |  |
| Mwamba 2018 | CLG5 | Zambia | Community adherence groups and dedicated VL clinic for unstable patients | 28  (27/97) |  |  |  |
| **ADHERENCE (%)** |  |  |  |  |  |  |  |
| Kagimu 2018 | CLG4 | Uganda | Community client led ART delivery |  | 95.0 (13/14)^a^ |  |  |
| **PRESCRIPTION REFILL (%)** |  |  |  |  |  |  |  |
| Avong 2018 | OFBIM5 | Nigeria | Community pharmacy ART dispensing |  | 100.0 (295/295) |  | - |
| Fox 2018 | HCWLG11 | South Africa | Adherence clubs |  | 92.0 (244/264) |  | 88.0 (250/284) |
| Okechukwu 2018 | CLG3 | Tanzania | Community ART refill groups |  | 97.9 (1,553/1,585)^a^ |  | 87.0  (11,973/13,762)^a^ |

a. Numerator calculated by authors

b. Rate was author calculated

c. Both numerator and denominator were author calculated based on other available data

d. National average

e. Cumulative incidence

ⱡ Updated data shared by the author

¥ Suppression defined as <400copies/mL

§ The comparator is a 2-month pick-up model in adherence clubs which is counted as a DSD model, rather than a SOC. However this manuscript was reported as having a comparator in the text.

### **Supplementary Table 8. Risk of bias assessment for cohort studies (Newcastle-Ottawa Scale*)**

| **Source** | **Model codes** | **Selection** | | | | **Comparability** | | **Outcome** | | | **Score** | **Quality** |
| --- | --- | --- | --- | --- | --- | --- | --- | --- | --- | --- | --- | --- |
|  |  | **1** | **2** | **3** | **4** | **1** | | **1** | **2** | **3** |  |  |
| Avong 2018 | OFBIM5 | * | - | * | - | - | * | | * | * | 5 | Moderate |
| Pasipamire 2018 | HCWLG2  CLG1  HCWLG2 | * | - | * | * | ** | * | | * | * | 8 | High |
| Pahad 2018 | HCWLG6 | - | - | * | - | - | * | | * | - | 3 | Low |
| Cassidy 2018 | HCWLG9 HCWLG10  FBIM4 | * | * | * | * | - | * | | * | * | 7 | High |
| Chibesakunda 2019 | OFBIM11 | * | - | * | - | - | * | | * | - | 4 | Moderate |
| Finci 2018 | HCWLG3 | - | - | * | * | - | * | | * | - | 4 | Moderate |
| Kagimu 2018 | CLG4 | - | - | - | - | - | - | | - | - | 0 | Low |
| Kamerhe 2018 | OFBIM2  FBIM1  HCWLG1 | * | - | * | * | - | * | | * | * | 6 | Moderate |
| Manganye 2018 | HCWLG4 | * | - | - | * | - | - | | * | - | 3 | Low |
| Mdala 2018 | FBIM3 | - | - | - | - | - | - | | * | - | 1 | Low |
| Meehan 2018 | HCWLG5 | * | - | * | * | - | * | | * | - | 4 | Moderate |
| Mothibi 2018 | OFBIM3 | * | - | - | * | - | - | | * | * | 4 | Moderate |
| Mulenga 2019 | OFBIM9 | * | - | - | * | - | - | | - | * | 3 | Low |
| Mwamba 2018 | CLG5 | - | - | * | * | - | * | | * | - | 4 | Moderate |
| Mwanda 2018 | OFBIM10 | - | - | - | - | - | - | | * | * | 2 | Low |
| Mwila 2018 | FBIM7 | - | - | - | - | - | - | | * | - | 1 | Low |
| Nwabueze 2018 | OFBIM6 | * | - | - | * | - | - | | * |  | 3 | Low |
| Okechukwu 2018 | CLG3 | * | * | - | * | - | - | | * | * | 5 | Moderate |
| Roy 2018 | HCWLG12 | * | * | * | * | - | * | | * | - | 6 | Moderate |
| Tchissambou 2018 | OFBIM1 | * | - | * | * | - | * | | * | * | 6 | Moderate |
| Nsumba 2019 | FBIM6 | - | - | - | * | - | - | | * | - | 2 | Low |
| Van Rompaey 2019 | CLG2 | * | - | * | * | - | * | | * | - | 5 | Moderate |

*The assessment was conducted using the Newcastle Ottawa Scale for cohort studies (<http://www.ohri.ca/programs/clinical_epidemiology/nosgen.pdf>). The items marked with a star (*) for each category are counted as one (“1”) and factored into the final scoring which ranges from zero to nine (lowest to highest). To simplify interpretation those studies that scored 7 or above were categorized as high quality, those scoring between 4-6 were of moderate quality, and those scoring below 4 were considered low quality.^17^

### **Supplementary Table 9. Risk of bias assessment for cluster randomized trials (Cochrane Collaboration’s tool)**

| **Source** | **Bias arising from the randomization process** | **Bias arising from the timing of identification and recruitment of individual participants in relation to timing of randomization** | **Bias due to deviations from intended interventions** | **Bias due to missing outcome data** | **Bias in measurement of the outcome** | **Bias in selection of the reported result** | **Overall bias** |
| --- | --- | --- | --- | --- | --- | --- | --- |
| Fox 2019 and Fox 2018 | Low risk | Low risk | Low risk | Low risk | Low risk | Low risk | Low risk |
| Geldsetzer 2018 | Low risk | Low risk | Low risk | Low risk | Low risk | Low risk | Low risk |
| Havlir 2019 | Low risk | Low risk | Low risk | Low risk | Low risk | Low risk | Low risk |
| Lebelo 2019 | Low risk | Some concern | Some concern | Low risk | Low risk | Low risk | Some concern |
| Limbada 2019 | Low risk | Low risk | Low risk | Some concern | Low risk | Low risk | Some concern |
